# What causes extended layering of ionic liquids on the mica surface?†
†Electronic supplementary information (ESI) available: Additional AFM images. See DOI: 10.1039/c5sc00832h



**DOI:** 10.1039/c5sc00832h

**Published:** 2015-04-20

**Authors:** Xiao Gong, Andrew Kozbial, Lei Li

**Affiliations:** a Department of Chemical & Petroleum Engineering , Swanson School of Engineering , University of Pittsburgh , Pittsburgh , PA 15261 , USA; b Department of Mechanical Engineering & Materials Science , Swanson School of Engineering , University of Pittsburgh , Pittsburgh , PA 15261 , USA . Email: lel55@pitt.edu

## Abstract

Extended layering of ionic liquids (ILs) on the mica surface has been reported by several groups previously and it is generally accepted that the electrostatic interaction at the IL/mica interface is critical to the observed extended layering. Here we report that, indeed, water adsorption on the mica surface is the key to the extended layering of ionic liquids. The atomic force microscopy (AFM), attenuated total reflectance-fourier transform infrared spectroscopy (ATR-FTIR) and contact angle (CA) results show that ionic liquids form extended layering on a mica surface under ambient conditions when water is adsorbed on the mica surface under such conditions. However, when airborne hydrocarbon contaminants replace the water on the mica surface at the elevated temperatures, instead of layering, ionic liquids exhibit droplet structure, *i.e.*, dewetting. Based on the experimental results, we propose that water enables ion exchange between K+ and the cations of ILs on the mica surface and thus triggers the ordered packing of cations/anions in ILs, resulting in extended layering.

## Abstract 2

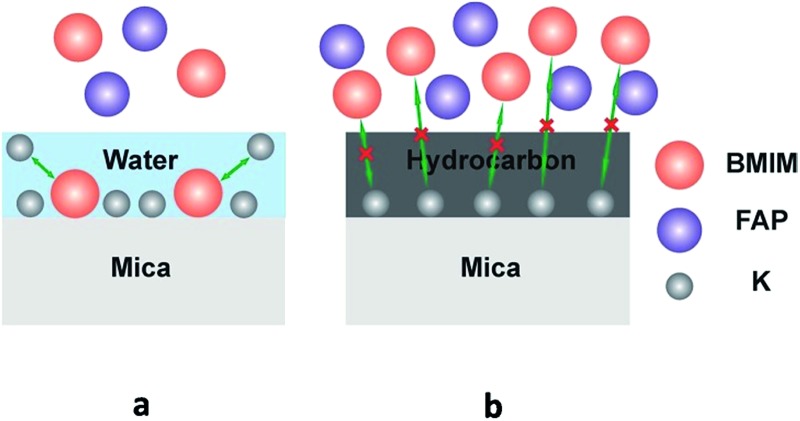
The adsorbed water on the mica surface is the key to the extended layering of ILs.

## Introduction

Ionic liquids (ILs) have attracted extensive interests because of their exceptional physicochemical properties and many promising applications.^1,2^ Some important applications such as lubrication^3^ and catalysis^4^ involve nanometer-thick IL films confined to solid surfaces. Therefore, it is critical to understand the molecular-level structure of ILs at the IL/solid interface. To date, ILs confined to many solid surfaces have been studied, including silica, mica, highly oriented pyrolitic graphite (HOPG), and sapphire.^5–8^ Among those solids, mica is the most frequently utilized one and many previous studies on the structure of IL nanofilms deposited on the mica surface have been reported.^6,8–10^ Liu *et al.* reported that ionic liquids formed ordered layering structure on the mica surface based on AFM results.^6^ Bovio *et al.* studied IL/mica interface by AFM and found that layering of IL molecules could extend up to 50 nm above the solid surface.^8^ Yokota *et al.* also observed extended layering structures of ILs on the mica surface by frequency-modulation atomic force microscopy.^9^ By analyzing the force–distance profile obtained when an AFM tip approaching and retracting from the mica surface in ILs, Atkin *et al.* also concluded that there is an extended layering structure of ILs on the mica surface.^11^ Using a surface force apparatus (SFA), Perkin *et al.* reported that the strong coulombic interaction at the IL–mica interface led to an extended layering structure of ILs.^12–14^ Recent simulation and computational results also indicated that ILs form extended layering structure on the mica surface.^15,16^ Since mica surface is negatively charged,^12,14^ it is generally believed that the electrostatic interactions at ILs/mica interface is the key to the above-mentioned extended layering of ILs on the mica surface^6,12,13,15,17–20^ though the exact governing mechanism is still under debate.^17,21,22^ However, it has been shown recently that complete dewetting of ILs occurs on a clean mica surface under ultra-high vacuum, and no layering structure was observed.^23^ It is worthy of mentioning that the IL studied in this work,^23^ 1-butyl-3-methylimidazolium bis (trifluoromethylsulfonyl)imide, is exactly the same as in ref. 8 and 15 where the extended layering was reported on the mica surface. Clearly, the dramatic difference cannot be attributed to the different chemistry of ILs, indicating the existence of unknown mechanisms governing the extended layering of ILs confined to the mica surface. Therefore, it is critical to uncover the underlying mechanisms.

Here we report that the chemistry of the “contaminants”, *e.g.*, water or hydrocarbons, on the mica surface plays a key role in determining the molecular-level structure of ILs at the IL/mica interface. Our experimental results have showed that, when water molecules are adsorbed on the mica surface under ambient conditions, the extended layering structure of ILs is observed on the mica surface. Once airborne hydrocarbons replace the adsorbed water on the same mica surface, a droplet (dewetting) structure of ILs is observed. These results shed new light to the governing mechanisms of the extended layering structure of ILs confined to a charged solid surface and provide a new dimension in controlling the structure of ILs at the IL/solid interface.

## Results and discussion

### Results

**Fig. 1:**
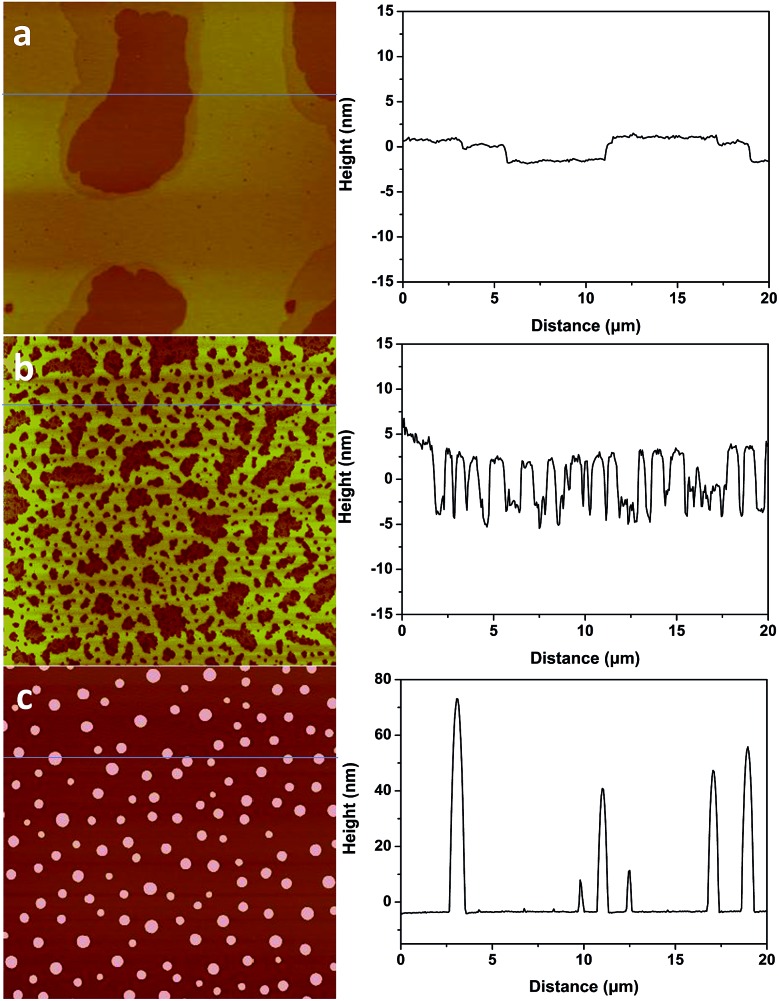
(left) AFM images of BMIM-FAP (1 g L^–1^ solution) dip-coated on a freshly cleaved mica surface (a), mica heated for 5 min at 120 °C (b), and mica heated for 1 h at 120 °C (c). AFM images are 20 μm × 20 μm and height bar is 20 nm. (right) Corresponding line profiles.

1-Butyl-3-methylimidazolium tris(pentafluoroethyl) trifluorophosphate (BMIM-FAP) (1 g L^–1^ solution in 2,3-dihydrodecafluoropentane) was applied on atomically smooth mica by dip-coating and the detailed procedure was described elsewhere^24^ (22 °C, RH = 45%). The topography of BMIM-FAP/mica was characterized by atomic force microscopy (AFM). For the sample fabricated with freshly cleaved mica, the extended layering structure is clearly visible as shown in Fig. 1a and no droplet is observed. The thickness of the thin layer is ∼0.85 nm which is consistent with the reported molecular diameter of BMIM-FAP (0.84 nm) determined from the bulk density.^25^ Indeed, even when the concentration of the IL solution is as high as 5 g L^–1^, the extended layering structure is still observed and the layering structure extends to above 17 nm in this case (Fig. S1†). These observations are consistent with previous reports showing extended layering of ILs on the mica surface.^6,8,9^ Interestingly, when the freshly cleaved mica was heated in a conventional oven at 120 °C for 5 min before dip-coating, a more “dewetting-like” network structure was observed (Fig. 1b). If the freshly cleaved mica was heated at 120 °C for 1 h before dip-coating, the droplets were observed on the BMIM-FAP/mica sample (Fig. 1c). Those droplets were 10–80 nm in height and 300–900 nm in diameter. It is worth noting that, even when the concentration of the IL solution is as low as 0.001 g L^–1^, the droplets were still observed (Fig. S2†), indicating complete dewetting of ILs. It was also found that, for the BMIM-FAP/mica sample fabricated using freshly cleaved mica, the droplet structure is observed when the humidity in the lab is low, *i.e.*, RH < 30% (Fig. S3†).

### Discussions

**Fig. 2:**
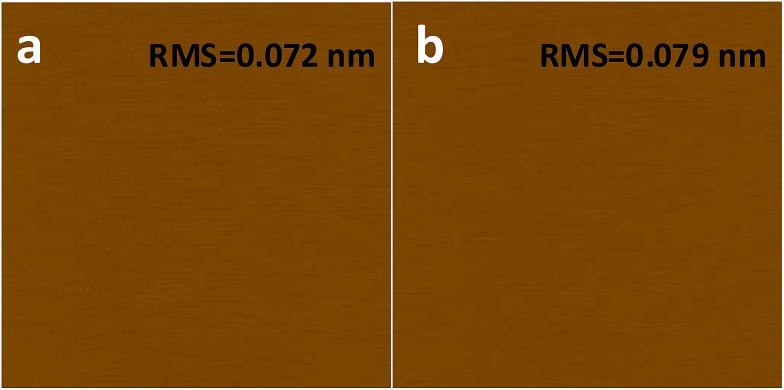
AFM images of freshly cleaved (a) and heat treated (1 h at 120 °C) mica (b). AFM images are 2 μm × 2 μm, and the height bar is 10 nm.

**Fig. 3:**
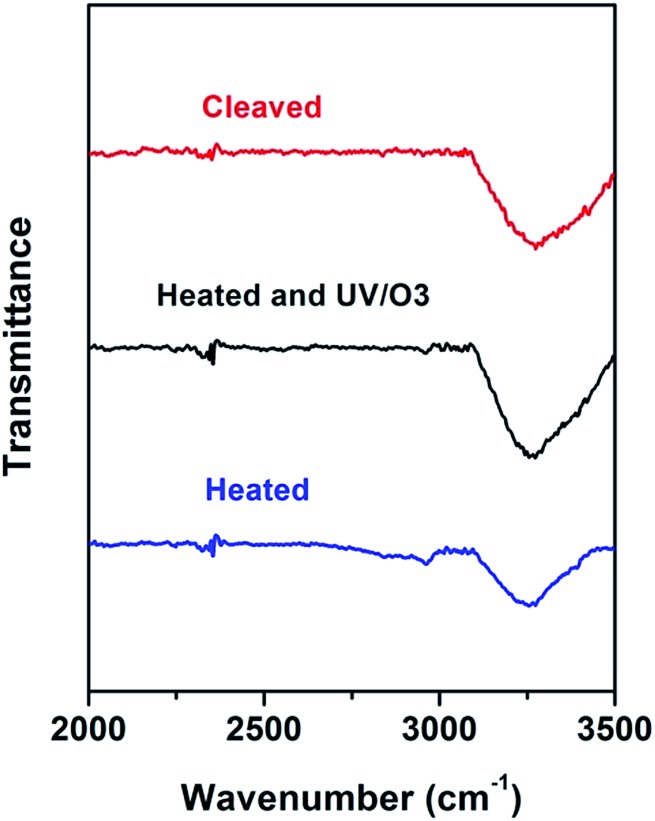
ATR-FTIR of freshly cleaved mica (red), heat-treated (1 h at 120 °C) mica (blue), and heat-treated mica with UV/O3 treatment (black).

What has changed on the mica surface after heating? Since mica has high thermal stability,^26,27^ it is unlikely there is chemical change of the mica itself at 120 °C for 1 h. Another possibility is the increase in the roughness since it has been reported that the layering of liquids will not occur at the liquid/solid interface when the solid surface is rough.^28^ However, AFM results (Fig. 2) showed that there is almost no change in roughness before and after heating. To understand the change of the mica surface before and after heating at 120 °C/1 h, the mica surface was characterized by ATR-FTIR. As shown in Fig. 3, before heating, there is a broad peak between 3000 cm^–1^and 3500 cm^–1^ that can be attributed to the adsorbed water.^29^ After heating, the intensity of the “water” peak decreases significantly while new peaks located around 2845 cm^–1^ and 2960 cm^–1^, which are attributed to the CH_2_ and CH_3_ moiety,^30,31^ show up. It has been well documented that water adsorbed on the mica surface can be up to 1 nm^32–34^ at high humidity and low temperature. Even at room temperature and ambient conditions, the adsorbed water is ∼0.4 nm.^35^ Therefore, the observed “water” peak in ATR-FTIR spectrum is attributed to the adsorbed water. Since water molecules can be removed at 120 °C, some airborne hydrocarbon contaminants will replace the water after heating, as indicated by the “C–H” peaks observed for the sample after heating. It should be noted that, in the current study, the adsorbed water and/or hydrocarbon molecules cannot be detected by AFM (Fig. 2), which is attributed to their high mobility under ambient conditions as reported in previous research.^35^

If the above-mentioned hypothesis is correct, it is expected that the intensity of “C–H” peaks will decrease and the intensity of “water” peak will increase after the removal of airborne hydrocarbons. Since it is well known that UV/O3 treatment removes the airborne hydrocarbons effectively,^36^ UV/O3 treatment was conducted to test the hypothesis. As shown in Fig. 3, after 30 minute UV/O3 treatment of the heated sample, the “C–H” peaks almost disappear while the intensity of the “water” peak increases again. The FTIR results suggested that there is a competition in the adsorption of water and airborne hydrocarbons on the mica surface. Under ambient conditions, water is preferably adsorbed on the mica surface. At the elevated temperature, the water is replaced by some airborne hydrocarbons, which should be high-boiling point volatile organic chemicals (VOCs). Based on the previous report^37^ on the VOCs in the local area, some possible candidates are perchloroethylene, ethylbenzene, alpha-pinene, hexanal, *p*-xylene, *m*-xylene, and *o*-xylene.

**Fig. 4:**
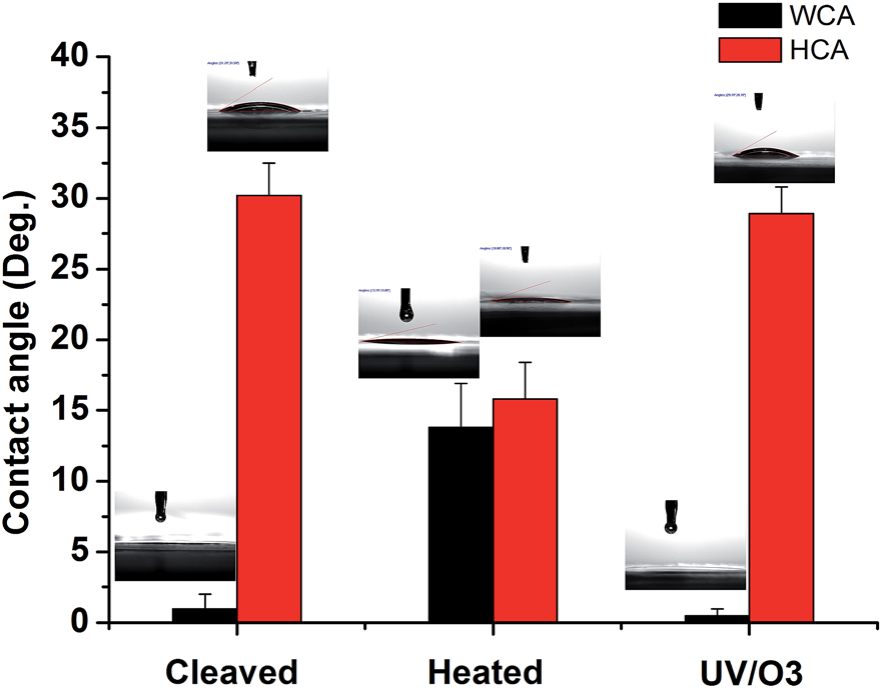
Water contact angle (WCA) and hexadecane contact angle (HCA) on freshly cleaved mica, heat-treated (1 h at 120 °C) mica, and heat-treated mica with UV/O3 treatment, respectively.

To further test the hypothesis, contact angle (CA) tests were conducted on mica surfaces with different treatments and the results are shown in Fig. 4. The water contact angle (WCA) on freshly cleaved mica was almost 0° and hexadecane contact angle (HCA) was ∼30.2 ± 2.3°. The result is in line with the picture that there are adsorbed water molecules on the mica surface so that WCA is very low and HCA is relatively high. Indeed, previous research showed that the oil contact angle on the glass surface with adsorbed water is ∼37° while the WCA on the same surface is around 0°.^38^ After heat treatment, the WCA on mica increased to 13.8 ± 3.1° while the HCA decreased to 15.8 ± 2.6°, indicating that the water is (partially) replace by some airborne hydrocarbons. Once the heated mica was treated by UV/O3, the WCA became 0° again and the HCA was ∼29.1 ± 1.9°, which can be explained by the (partial) removal of airborne hydrocarbons followed by the re-adsorption of water. The CA results are consistent with the ATR-FTIR results and supported the above-mentioned hypothesis.

The FTIR and WCA results indicated that when there is water on the mica surface, the BMIM-FAP exhibits the extended layering structure. When the water is replaced by some airborne hydrocarbons, the BMIM-FPA has droplet (dewetting) structure on the mica surface. What are the underlying mechanisms?

**Fig. 5:**
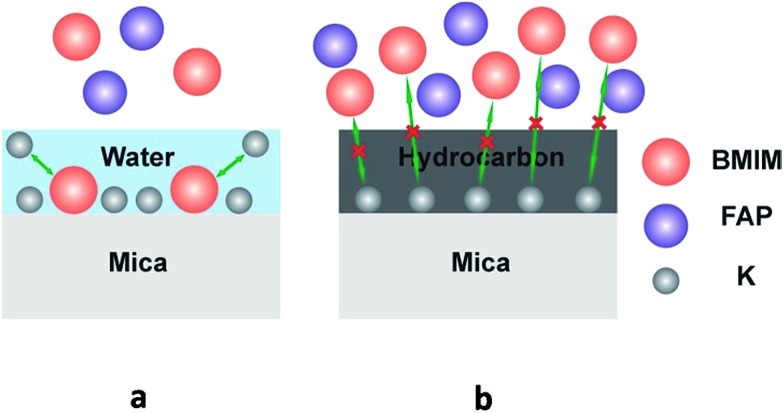
Schematic of ions exchange at the freshly cleaved mica surface (a), and schematic of ions exchange blocked at the heated mica surface (b).

It has been reported^39–41^ that dissociation of surface K+ ions will occur if the freshly cleaved mica is in contact with an effective electrolyte, *i.e.*, liquids with high dielectric constant such as water whose dielectric constant is 80.4. However, BMIM-FAP has a dielectric constant estimated to be lower than 15 and is not an effective electrolyte.^42^ Therefore, only when there is a layer of water on the mica surface, K+ ions will leave the mica surface and thus the surface will carry negative charges (Fig. 5a). As a result, the cations of ionic liquids are able to occupy the “empty” site, initiating the ordered packing of cations/anions of the ILs, *i.e.*, the layering structure. When the water is replaced by airborne hydrocarbons, the dissociation of the surface K+ ions becomes impossible and the ionic liquids do not wet the hydrocarbon-covered mica (Fig. 5b). As a result, the dewetting occurs and the droplet was observed. Our results also explain the previous controversy initiated by the observed “dewetting” behavior of ILs on the mica surface under high vaccum.^23^ Majority of previous reports on IL/Mica were conducted under ambient conditions. Therefore, water adsorption on the mica surface is expected and that is why layering of ILs at the IL/mica interface has been frequently reported. For the only study^23^ conducted in the high vacuum, there is no water adsorption and, therefore, dewetting was observed.

## Experimental

### Sample preparation

The ionic liquids were dissolved in the 2,3-dihydrodecafluoropentane and applied on the mica by dip-coating based on a previously established “dip-withdraw” procedure in our lab,^24^ with a KSV-DCX2 dip-coater equipped with a Kinetic Systems vibration free platform at a withdraw rate of 1 mm second^–1^ at room temperature (RH = 45%).

### AFM

Tapping mode AFM images were acquired using a Veeco Dimention V AFM. Silicon AFM probes were utilized to image the samples in soft tapping mode.

### ATR-FTIR

Spectra were collected with a Bruker VERTEX-70LS FTIR and a Bruker Hyperion 2000 FTIR microscope in reflectance mode utilizing a germanium 20× ATR objective and a liquid nitrogen cooled mid-band MCT A detector (7000–600 cm^–1^ spectral range). Before measurements, the system was purged for 20 minutes with nitrogen gas and a background spectrum was collected without having the ATR crystal contacting the sample. Each sample spectrum was collected for 150 scans with a resolution of 4 cm^–1^ and a total acquisition time of 2.5 min.

### WCA and HCA measurement

Deionized (DI) water, produced from a Millipore Academic A10 system with total organic carbon below 40 ppb, and hexadecane (anhydrous, ≥99%, Sigma-Aldrich) were used as the testing liquids for contact angle measurements. The water contact angle (WCA) and hexadecane contact angle (HCA) measurement was conducted with a VCA optima XE contact angle system at room temperature. Each liquid droplet has a volume of ∼2 μL and was carefully introduced to the sample surface. A charge-coupled device (CCD) camera was used to take images of water or hexadecane droplets, which was followed by an automatic calculation of the static contact angle by the vendor-supplied software. Each static WCA and HCA measurement was repeated three times and the average value was reported.

### UV/O3 treatment

UV/O3 treatment was conducted with a BioForce Nanosciences UV/Ozone Procleaner. This cleaner emits a high-intensity UV light with the wavelengths of both 185 nm and 254 nm. All the UV treatment as conducted under near-constant temperature (∼22 °C) in the ambient air for 30 minutes.

## Conclusions

In conclusion, we presented an experimental study on the effect of surface adsorption of mica on the molecular-level structure of ILs at the IL/mica interface. Our results show that the water adsorption on the mica surface is the key to the extended layering structure of ILs on the mica surface. We proposed that water serves as an effective electrolyte and thus facilitates the ion exchange between K+ ions at the mica surface and the cations of ILs, which initiates the ordered packing of cations/anions of ILs. Our finding suggests that the chemistry of surface contaminants is a new dimension to manipulate the molecular-level structure of ILs at the IL/solid interface though further research is required to fully uncover the detailed mechanisms.
