# Supplementary material for: What causes extended layering of ionic liquids on the mica surface?
Source: Chem Sci. 2015 Apr 20;6(6):3478–82. doi: 10.1039/c5sc00832h (PMC5492871; doi:10.1039/c5sc00832h)
Supplement: Supplementary file 1 [file SC-006-C5SC00832H-s001.pdf]

Electronic Supplementary Material (ESI) for Chemical Science.
This journal is © The Royal Society of Chemistry 2015

Supporting Information

What Causes Extended Layering of Ionic liquids on the Mica Surface?

Xiao Gong[a], Andrew Kozbial[a], and Lei Li[a,b,*]

[a] Department of Chemical & Petroleum Engineering, Swanson School of Engineering, University of Pittsburgh, Pittsburgh, PA, USA

[b] Department of Mechanical Engineering & Materials Science, Swanson School of Engineering, University of Pittsburgh, Pittsburgh, PA, USA

Correspondence and requests for materials should be addressed to L.L. (email: lel55@pitt.edu).

Morphology of a "thick" BMIM FAP nanofilm on the fresh mica surface

Freshly cleaved mica was dip-coated with a 5 g/L BMIM FAP/Vertrel (2,3-dihydrodecafluoropentane) solution. Topography of the resulting BMIM FAP/mica sample was characterized by AFM. As shown in Figure S1, a very flat nanofilm was produced on the mica surface and the thickness of the nanofilm is around 17 nm.

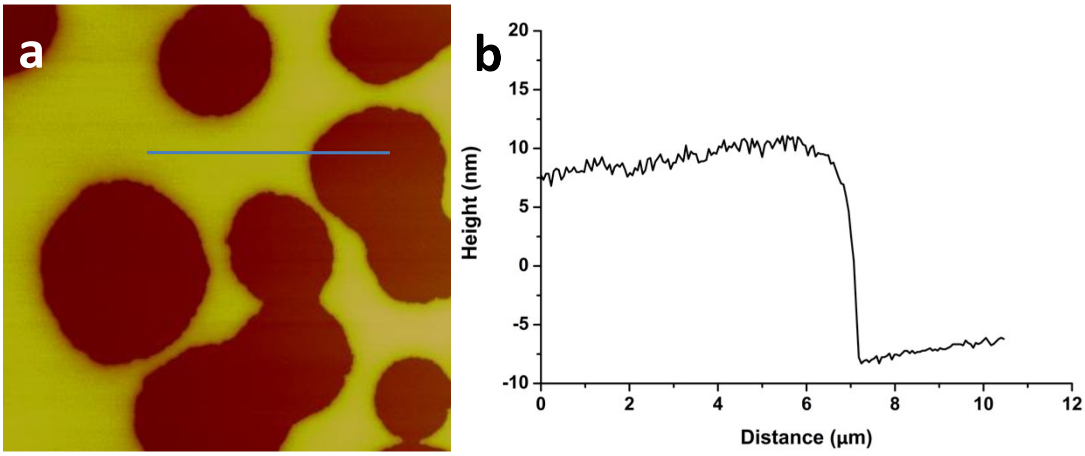

Figure S1. AFM image (a), and corresponding profile (b) of BMIM FAP dip-coated on a freshly cleaved mica. The concentration of BMIM FAP/vertrel solution is 5 g/L. AFM image is 20μm×20μm, and the height bar is 50 nm.

Morphology of BMIM FAP on the heated mica surface

Mica was cleaved and heat treated in an conventional oven at 120 °C for 1 h. Afterwards, the mica was dip-coated with 0.01 g/L and 0.001 g/L BMIM FAP/Vertrel (2,3-dihydrodecafluoropentane) solution, respectively. The average thickness of the resulting BMIM FAP nanofilms is estimated to be in the order of angstrom or lower[S1]. The topography of BMIM FAP/mica samples was characterized by AFM. As shown in Figure S2, for both 0.01 g/L and 0.001 g/L sample, very small droplets are clearly visible on the mica surface, indicating complete dewetting of BMIM FAP on the heated mica surface.

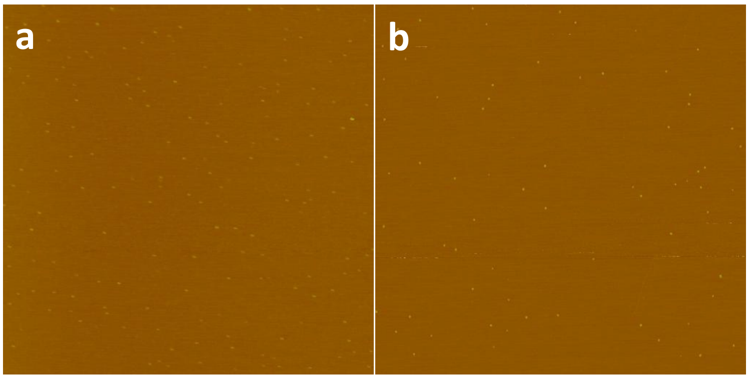

Figure S2. AFM images of BMIM FAP on the heat-treated (120 ℃ for 1 h) mica surface. The concentration of BMIM FAP/vertrel solution is 0.01 g/L (a), and 0.001 g/L (b), respectively. AFM images are 2μm×2μm, and the height bar is 10 nm.

Morphology of a BMIM FAP on the fresh mica surface under low RH

Freshly cleaved mica was dip-coated with a 1 g/L BMIM FAP/Vertrel (2,3-dihydrodecafluoropentane) solution at room temperature and low RH (= 20%). Topography of the resulting BMIM FAP/mica sample was characterized by AFM. As shown in Figure S3, droplets were produced on the fresh cleaved mica surface, indicating dewetting has occurred.

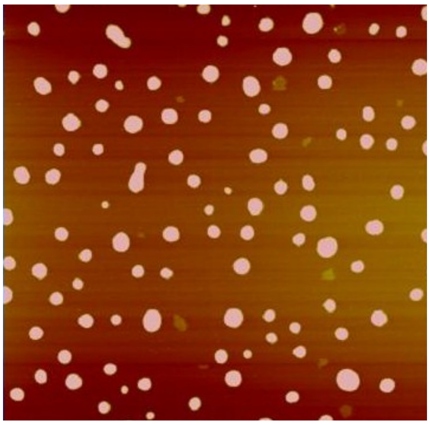

Figure S3. AFM image of BMIM-FAP dip-coated on a freshly cleaved mica at room temperature with RH = 20%. The concentration of BMIM FAP/vertrel solution is 1 g/L. AFM image is 20 μm×20 μm, and the height bar is 20 nm.

Reference:

S1. Merzlikine, A. G.; Li, L.; Jones, P.; Hsia, Y. *Tribol. Lett.* **2005**, *18*, 279.
